# Supplementary material for: Identification of heterosis and combining ability in the hybrids of male sterile and restorer sorghum [Sorghum bicolor (L.) Moench] lines
Source: PLoS One. 2024 Jan 2;19(1):e0296416. doi: 10.1371/journal.pone.0296416 (PMC10760902; doi:10.1371/journal.pone.0296416)
Supplement: S1 Fig — (PDF) [file pone.0296416.s001.pdf]

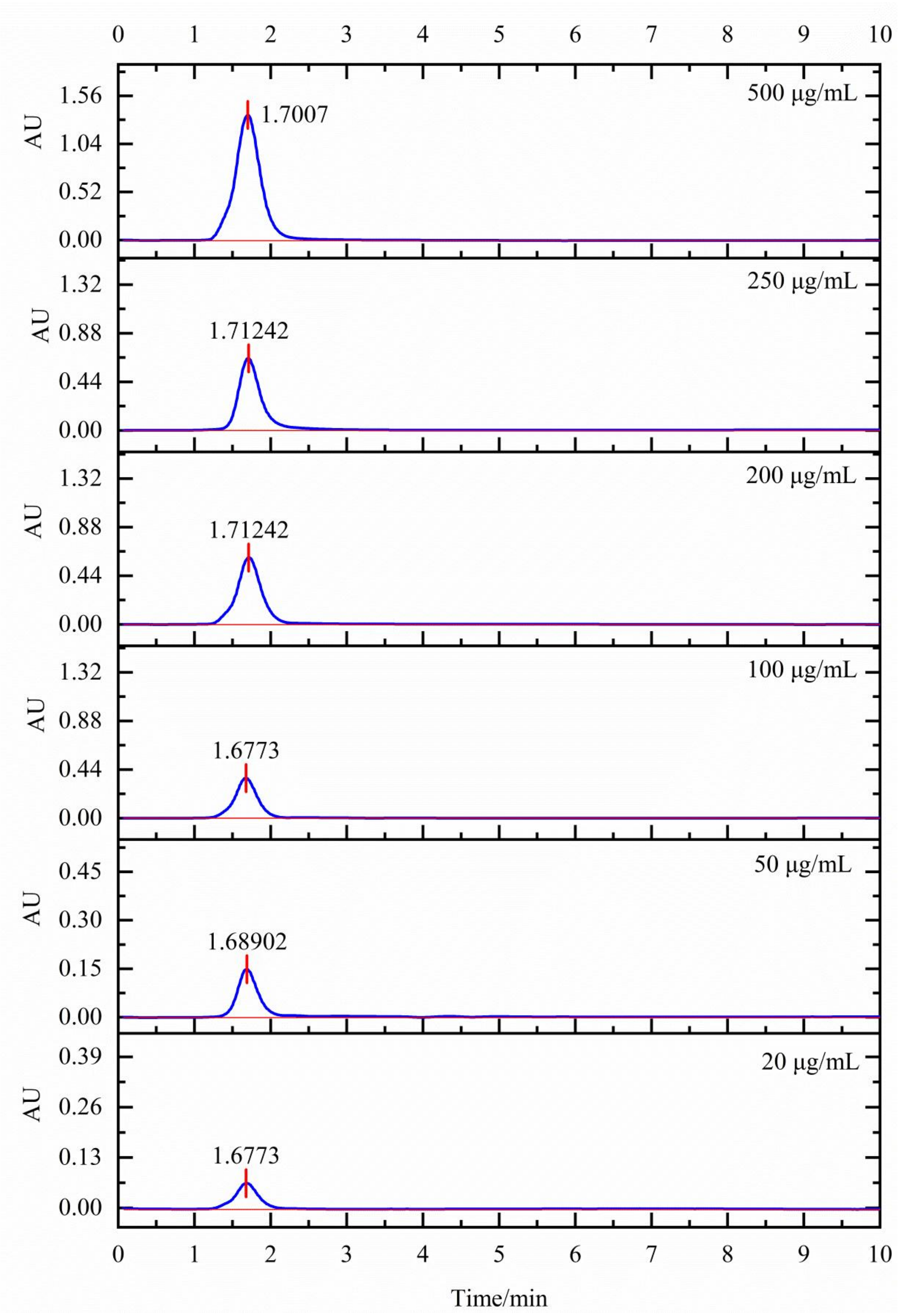

S1 Fig. HPLC chromatograms for the standard solutions of iristectorigenin A at six different concentrations.
