# Supplementary figures and images for: Identification of heterosis and combining ability in the hybrids of male sterile and restorer sorghum [Sorghum bicolor (L.) Moench] lines
Source: PLoS One. 2024 Jan 2;19(1):e0296416. doi: 10.1371/journal.pone.0296416 (PMC10760902; doi:10.1371/journal.pone.0296416)

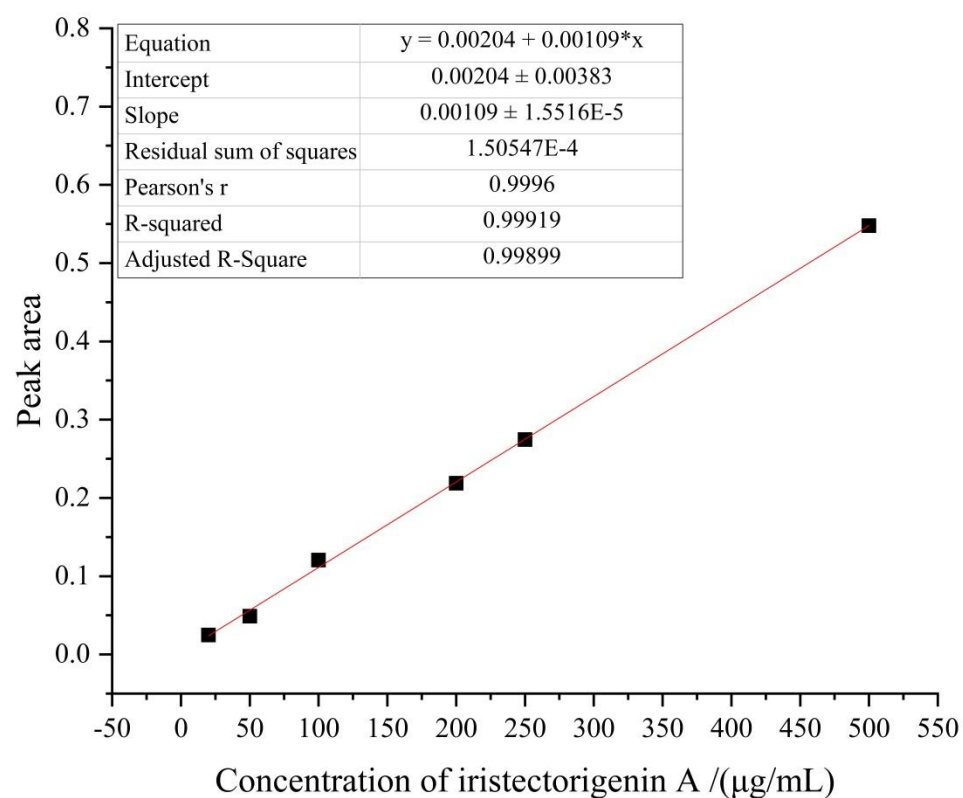

S2 Fig. The standard curve for iristectorigenin A used in this study.

Supplement: S2 Fig — (PDF) [file pone.0296416.s002.pdf]

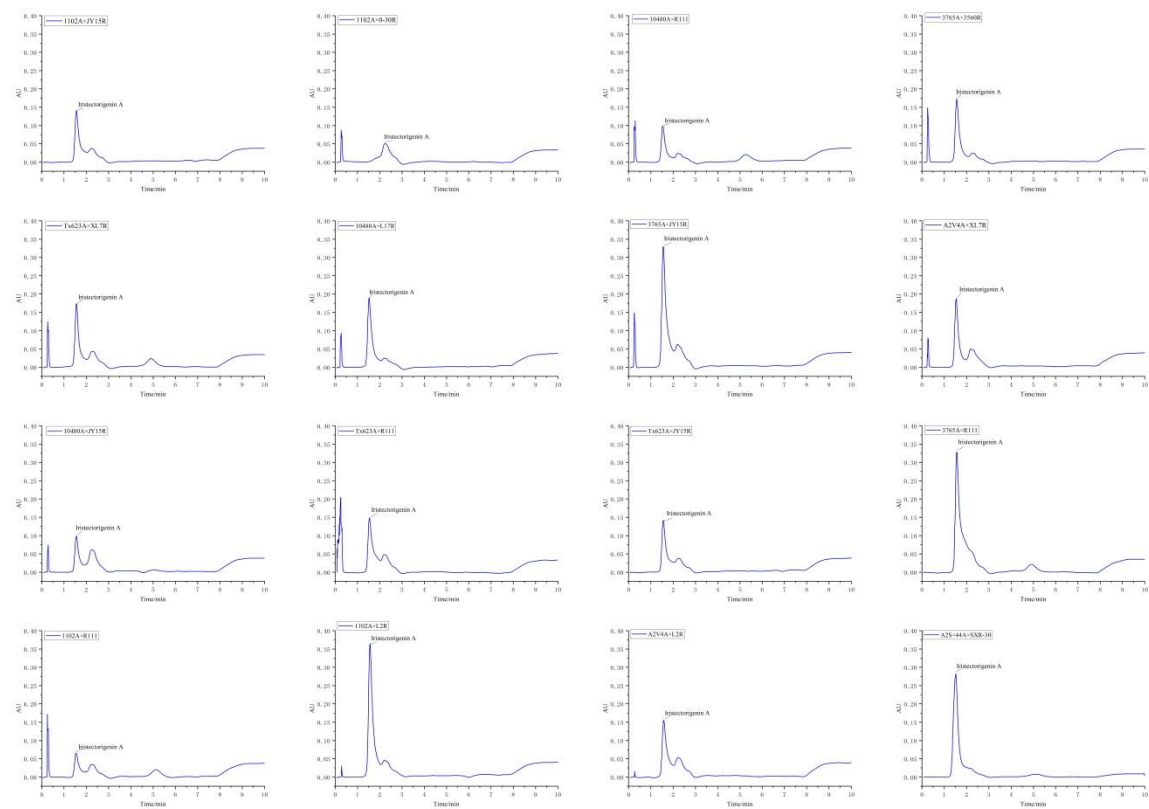

S3 Fig. All chromatograms of iristectorigenin A content of 15 heterotic hybrids

Supplement: S3 Fig — (PDF) [file pone.0296416.s003.pdf]
