# Supplementary material for: Identification of heterosis and combining ability in the hybrids of male sterile and restorer sorghum [Sorghum bicolor (L.) Moench] lines
Source: PLoS One. 2024 Jan 2;19(1):e0296416. doi: 10.1371/journal.pone.0296416 (PMC10760902; doi:10.1371/journal.pone.0296416)
Supplement: S1 Table — (PDF) [file pone.0296416.s004.pdf]

S1 Table. Estimates of specific combining ability effects (SCA) for measured characters

No.	Crosses	Plant height	Panicle length	Grain weight per Panicle	1000-grain weight	Grain yield
1	Tx3197A × 5-27R	5.89*	1.40	-8.88	0.53	0.24
2	Tx3197A × LZ615R	15.96**	1.22	19.25*	1.37	0.74
3	Tx3197A × SCSR	15.67**	0.54	18.14*	8.75**	0.37
4	Tx3197A × 0-30R	-7.56*	-0.24	6.29	-2.77	-0.39
5	Tx3197A × R111	14.55**	0.78	-2.79	3.86*	-0.17
6	Tx3197A × L17R	14.7**	1.35	-4.12	2.03	0.06
7	Tx3197A × L2R	4.15	-1.16	11.99	-0.8	0.14
8	Tx3197A × J12R	-9.45**	1.05	14.24	-0.23	0.37
9	Tx3197A × J105R	9.53**	-4.4**	0.16	1.41	-0.73
10	Tx3197A × XL7R	-15.18**	-3.83**	3.28	0.68	0.29
11	Tx3197A × JL5R	-13.18**	0.81	-15.27	-1.94	0.76
12	Tx3197A × 1383-2R	-2.76	3.34**	-11.2	-1.77	-0.18
13	Tx3197A × 3560R	-9.04**	-2.95**	-17.57*	-4.81*	-0.84*
14	Tx3197A × JY15R	-23.28**	2.09*	-13.51	-6.32**	-0.67
15	L407A × 5-27R	4.54	-0.01	8.87	-1.75	0.72
16	L407A × LZ615R	4.78	-0.11	35.91**	0.96	0.4
17	L407A × SCSR	-10.84**	1.54	-2.86	-1.46	-0.11
18	L407A × 0-30R	10.59**	0.97	2.9	2.62	0.22
19	L407A × R111	-4.29	1.46	-12.68	-3.82	-0.18
20	L407A × L17R	-29.48**	-2.98**	-3.03	0.38	-0.56
21	L407A × L2R	5.30	-2.82**	-24.82**	-0.21	-0.67
22	L407A × J12R	-12.62**	-2.94**	-17.8*	1.46	-0.08
23	L407A × J105R	11.69**	1.94*	17.43*	3.09	0.42
24	L407A × XL7R	2.97	1.81	2.55	3.6	0.01
25	L407A × JL5R	17.97**	-0.52	8.56	-5.72**	-0.25
26	L407A × 1383-2R	-1.60	0.01	1.53	2.28	0.37
27	L407A × 3560R	7.11*	1.56	-11.11	-1.23	0.23
28	L407A × JY15R	-6.12*	0.09	-5.45	-0.2	-0.51
29	A2V4A × 5-27R	-12.73**	-0.98	-9.39	-3.85	0.02
30	A2V4A × LZ615R	-4.66	1.57	21.6**	4.82*	0.27
31	A2V4A × SCSR	13.05**	2.99**	2.93	-1.73	0.31
32	A2V4A × 0-30R	0.15	-3.25**	-8.86	-4.18*	-1.12**
33	A2V4A × R111	-4.06	1.00	-9.77	-4.18*	-0.57
34	A2V4A × L17R	-19.92**	-1.97*	-9.54	-4.82*	-0.61

No.	Crosses	Plant height	Panicle length	Grain weight per Panicle	1000-grain weight	Grain yield
35	A2V4A × L2R	20.86**	1.52	25.27**	2.52	1.55**
36	A2V4A × J12R	18.27**	-0.13	-14.84	-0.54	-0.03
37	A2V4A × J105R	-6.42*	-0.52	-6.28	-0.97	-0.27
38	A2V4A × XL7R	4.53	-1.52	5.31	1.06	1.61**
39	A2V4A × JL5R	11.2**	-0.84	-11.08	5.48**	-0.38
40	A2V4A × 1383-2R	-5.37	-0.15	12.89	2.22	0.4
41	A2V4A × 3560R	-3.99	2.17*	11.92	3.57	-0.23
42	A2V4A × JY15R	-10.9**	0.10	-10.16	0.6	-0.95*
43	1102A × 5-27R	4.03	3.52**	33.74**	1.75	-0.62
44	1102A × LZ615R	4.10	0.10	-25.3**	-1.51	-0.65
45	1102A × SCSR	9.82**	-1.28	2.49	2.6	0.05
46	1102A × 0-30R	4.58	-0.52	7.04	1.42	1.86**
47	1102A × R111	-5.97*	1.37	-0.3	-0.18	0.29
48	1102A × L17R	-8.49**	-2.34*	-8.59	-0.09	-0.98*
49	1102A × L2R	-9.04**	-1.08	9.57	-1.28	1.81**
50	1102A × J12R	20.36**	0.90	2.66	1.49	-0.18
51	1102A × J105R	-7.66**	2.22*	-6.89	-5.91**	0.62
52	1102A × XL7R	8.96**	-3.02**	2.37	0.46	-1.69**
53	1102A × JL5R	-21.37**	-2.51**	20.21**	4.81**	-0.85*
54	1102A × 1383-2R	8.72**	0.52	-36.04**	-7.22**	-0.53
55	1102A × 3560R	-4.56	-0.06	-8.57	-2.83	-0.59
56	1102A × JY15R	-3.47	2.17*	7.61	6.46**	1.46**
57	10480A × 5-27R	2.41	-3.38**	-7.21	0.89	-0.06
58	10480A × LZ615R	2.48	-0.57	-34.19**	-3.6	-0.38
59	10480A × SCSR	-9.14**	-3.25**	-14.17	-3.22	0.05
60	10480A × 0-30R	2.96	0.85	-2.36	-1.98	0.68
61	10480A × R111	-3.26	-0.67	13.33	1.06	-0.54
62	10480A × L17R	22.55**	3.56**	23.1**	3.62	1.81**
63	10480A × L2R	-5.66	2.72**	-25.66**	-2.84	-1.34**
64	10480A × J12R	9.41**	0.93	20.66**	4.03	0.04
65	10480A × J105R	2.39	-3.18**	-5.18	2.27	0.08
66	10480A × XL7R	-17.99**	2.69**	0.72	-0.79	0.01
67	10480A × JL5R	-15.33**	2.2*	-14.61	-1.05	0.16
68	10480A × 1383-2R	2.44	-0.45	22.04**	4.92*	-0.25
69	10480A × 3560R	7.48*	-0.73	5.87	-1.59	-0.46

No.	Crosses	Plant height	Panicle length	Grain weight per Panicle	1000-grain weight	Grain yield
70	10480A × JY15R	-0.76	-0.73	17.64*	-1.73	0.2
71	Tx623A × 5-27R	-1.93	-2.47**	-13.08	-0.04	-0.12
72	Tx623A × LZ615R	19.47**	-2.69**	7.48	2.7	0.5
73	Tx623A × SCSR	-7.15*	0.17	-9.53	-2.42	0.11
74	Tx623A × 0-30R	-10.72**	3.93**	7.39	1.5	-0.83*
75	Tx623A × R111	-1.93	0.41	20.21**	0.96	0.66
76	Tx623A × L17	15.21**	2.51**	-12.06	-3.54	0.26
77	Tx623A × L2R	-5.34	-1.16	-10.41	2.64	-0.75
78	Tx623A × J12R	-26.43**	-1.95*	-5.89	-6.13**	-0.61
79	Tx623A × J105R	-8.29**	2.73**	-15.27	-4.89*	0.03
80	Tx623A × XL7R	-0.67	1.60	-0.08	0.88	1.1**
81	Tx623A × JL5R	8.66**	0.44	6.66	-1.37	0.27
82	Tx623A × 1383-2R	0.42	-2.03*	5.84	0.16	-0.52
83	Tx623A × 3560R	-13.2**	1.35	5.43	4.72*	-0.19
84	Tx623A × JY15R	31.9**	-2.85**	13.32	4.81*	0.09
85	3765A × 5-27R	-2.21	1.9*	-4.05	2.46	-0.17
86	3765A × LZ615R	-42.14**	0.48	-24.75**	-4.74*	-0.88*
87	3765A × SCSR	-11.42**	-0.70	3.01	-2.52	-0.78
88	3765A × 0-30R	0.01	-1.74	-12.4	3.39	-0.42
89	3765A × R111	4.96	-4.35**	-8.01	2.29	0.52
90	3765A × L17R	5.44	-0.14	14.23	2.42	0.02
91	3765A × L2R	-10.28**	1.97*	14.05	-0.04	-0.74
92	3765A × J12R	0.46	2.15*	0.97	-0.1	0.49
93	3765A × J105R	-1.23	1.20	16.03	5	-0.15
94	3765A × XL7R	17.39**	2.27*	-14.15	-5.89**	-1.33
95	3765A × JL5R	12.05**	0.41	5.53	-0.21	0.3
96	3765A × 1383-2R	-1.85	-1.23	4.94	-0.61	0.7
97	3765A × 3560R	16.2**	-1.35	14.03	2.15	2.07**
98	3765A × JY15R	12.63**	-0.88	-9.45	-3.63	0.38
	**SE (sij)**	5.781	1.853	16.709	3.832	0.786
	**SE (sij-skl)**	7.625	2.444	22.039	5.055	1.036

* significant at 5% level, ** significant at 1% level.
