# Supplementary material for: Identification of heterosis and combining ability in the hybrids of male sterile and restorer sorghum [Sorghum bicolor (L.) Moench] lines
Source: PLoS One. 2024 Jan 2;19(1):e0296416. doi: 10.1371/journal.pone.0296416 (PMC10760902; doi:10.1371/journal.pone.0296416)
Supplement: S2 Table — (PDF) [file pone.0296416.s005.pdf]

S2 Table. Average performance of parents related traits (2019-2020).

No.	Parents and hybrids	Plant height (cm)	Panicle length (cm)	Grain weight per Panicle	1000-grain weight
A1	Tx3197A	106.7	24.2	48.4	27.9
A2	L407A	96.0	27.0	32.5	24.4
A3	A_2V4A	120.3	23.2	51.0	25.4
A4	1102A	124.0	28.9	76.1	34.1
A5	10480A	144.0	34.4	75.5	23.3
A6	Tx623A	113.3	29.2	44.1	32.4
A7	3765A	124.4	27.4	74.4	35.1
R1	5-27R	112.7	26.7	51.0	33.1
R2	LZ615R	124.3	30.2	79.5	35.3
R3	SCSR	126.7	19.7	43.1	28.1
R4	0-30R	127.7	19.8	80.4	39.2
R5	R111	130.7	21.7	84.4	33.0
R6	L17R	127.3	26.1	51.0	36.3
R7	L2R	142.7	21.8	47.7	29.5
R8	J12R	109.3	28.6	37.0	25.0
R9	J105R	129.4	35.6	60.1	21.0
R10	XL7R	118.0	25.0	64.5	38.6
R11	JL5R	155.7	24.8	81.7	22.9
R12	1383-2R	148.7	26.7	77.8	26.0
R13	3560R	154.0	26.3	60.1	30.3
R14	JY15R	142.3	21.2	81.9	30.9
