# Supplementary material for: Identification of heterosis and combining ability in the hybrids of male sterile and restorer sorghum [Sorghum bicolor (L.) Moench] lines
Source: PLoS One. 2024 Jan 2;19(1):e0296416. doi: 10.1371/journal.pone.0296416 (PMC10760902; doi:10.1371/journal.pone.0296416)
Supplement: S3 Table — (PDF) [file pone.0296416.s006.pdf]

S3 Table. Average performance of hybrids related traits (2019-2020).

No.	Parents and hybrids	Plant height (cm)	Panicle length(cm)	Grain weight per Panicle(g)	1000-grain weight(g)
H1	Tx3197A × 5-27R	141.0	27.3	38.9	27.2
H2	Tx3197A × LZ615R	156.7	32.3	72.9	27.3
H3	Tx3197A × SCSR	161.0	23.7	51.9	33.7
H4	Tx3197A × 0-30R	146.7	24.3	68.5	31.6
H5	Tx3197A × R111	179.7	27.0	57.9	36.2
H6	Tx3197A × L17R	162.0	25.0	44.8	30.1
H7	Tx3197A × L2R	172.0	23.3	73.3	28.0
H8	Tx3197A × J12R	124.3	31.0	58.6	29.7
H9	Tx3197A × J105R	165.3	33.0	60.6	28.0
H10	Tx3197A × XL7R	175.3	28.0	54.0	34.8
H11	Tx3197A × JL5R	147.7	29.0	60.6	33.9
H12	Tx3197A × 1383-2R	149.7	29.3	82.5	31.4
H13	Tx3197A × 3560R	163.7	25.0	35.7	22.7
H14	Tx3197A × JY15R	171.0	27.7	57.5	25.7
H15	L407A × 5-27R	142.0	31.7	93.1	30.3
H16	L407A × LZ615R	188.0	33.3	98.3	31.6
H17	L407A × SCSR	193.0	30.3	90.8	25.9
H18	L407A × 0-30R	177.3	34.0	91.1	34.0
H19	L407A × R111	181.3	31.3	96.3	34.1
H20	L407A × L17R	152.3	28.3	74.8	29.9
H21	L407A × L2R	194.0	24.7	66.4	26.3
H22	L407A × J12R	146.0	32.7	64.9	22.8
H23	L407A × J105R	192.3	35.3	106.2	29.6
H24	L407A × XL7R	185.3	33.7	94.3	31.6
H25	L407A × JL5R	208.7	31.7	66.7	29.0
H26	L407A × 1383-2R	191.3	34.0	101.0	29.5
H27	L407A × 3560R	201.7	35.0	98.0	29.0
H28	L407A × JY15R	220.3	25.7	114.8	34.7
H29	A2V4A × 5-27R	161.3	29.7	83.7	27.9
H30	A2V4A × LZ615R	194.3	28.3	102.3	33.1
H31	A2V4A × SCSR	186.7	27.3	80.8	32.8
H32	A2V4A × 0-30R	188.0	27.7	81.0	35.3
H33	A2V4A × R111	209.3	30.0	79.1	36.1
H34	A2V4A × L17R	174.7	28.7	109.2	32.0

No.	Parents and hybrids	Plant height (cm)	Panicle length(cm)	Grain weight per Panicle(g)	1000-grain weight(g)
H35	A2V4A × L2R	218.0	28.0	105.3	31.7
H36	A2V4A × J12R	175.7	29.7	67.7	24.3
H37	A2V4A × J105R	178.3	32.0	129.4	30.2
H38	A2V4A × XL7R	193.7	27.3	92.7	30.9
H39	A2V4A × JL5R	222.0	27.0	103.6	33.8
H40	A2V4A × 1383-2R	212.3	30.0	98.1	34.3
H41	A2V4A × 3560R	190.0	28.7	61.2	23.7
H42	A2V4A × JY15R	211.7	30.0	112.9	40.7
H43	1102A × 5-27R	149.0	30.0	74.4	29.5
H44	1102A × LZ615R	189.0	29.5	58.0	38.7
H45	1102A × SCSR	167.0	29.7	73.1	27.1
H46	1102A × 0-30R	184.3	32.7	87.9	25.5
H47	1102A × R111	179.7	33.0	93.8	34.8
H48	1102A × L17R	162.0	29.7	87.4	30.4
H49	1102A × L2R	177.3	27.2	103.7	28.1
H50	1102A × J12R	168.7	33.0	87.2	26.9
H51	1102A × J105R	180.7	33.7	115.8	28.7
H52	1102A × XL7R	199.0	29.7	98.0	32.8
H53	1102A × JL5R	203.3	31.0	81.4	29.5
H54	1102A × 1383-2R	192.0	31.0	46.2	18.5
H55	1102A × 3560R	187.0	31.7	84.1	28.7
H56	1102A × JY15R	203.0	29.7	91.9	31.8
H57	10480A × 5-27R	146.0	33.0	88.9	25.6
H58	10480A × LZ615R	186.3	35.3	80.3	29.4
H59	10480A × SCSR	174.7	33.0	70.4	21.0
H60	10480A × 0-30R	174.0	32.7	112.4	28.3
H61	10480A × R111	180.3	34.3	102.9	30.7
H62	10480A × L17R	177.7	32.0	117.2	34.4
H63	10480A × L2R	168.3	34.5	118.0	31.2
H64	10480A × J12R	157.3	40.8	76.2	21.4
H65	10480A × J105R	194.7	34.7	110.8	31.2
H66	10480A × XL7R	169.3	36.7	93.7	30.1
H67	10480A × JL5R	174.0	31.3	68.4	19.7
H68	10480A × 1383-2R	201.7	30.0	83.3	23.7
H69	10480A × 3560R	175.0	24.5	58.5	18.9

No.	Parents and hybrids	Plant height (cm)	Panicle length(cm)	Grain weight per Panicle(g)	1000-grain weight(g)
H70	10480A × JY15R	181.7	29.2	91.7	28.0
H71	Tx623A × 5-27R	157.0	29.7	70.2	32.8
H72	Tx623A × LZ615R	184.7	36.0	96.0	24.1
H73	Tx623A × SCSR	174.0	31.3	78.0	26.6
H74	Tx623A × 0-30R	174.3	35.0	98.4	42.9
H75	Tx623A × R111	182.0	31.3	83.2	29.7
H76	Tx623A × L17	189.0	26.7	57.1	26.7
H77	Tx623A × L2R	157.0	30.3	53.1	30.7
H78	Tx623A × J12R	140.3	34.0	64.1	20.9
H79	Tx623A × J105R	202.3	35.0	92.7	28.8
H80	Tx623A × XL7R	172.0	31.3	70.7	22.3
H81	Tx623A × JL5R	159.0	32.3	98.5	34.4
H82	Tx623A × 1383-2R	189.0	29.3	79.9	26.3
H83	Tx623A × 3560R	231.7	33.3	83.2	33.2
H84	Tx623A × JY15R	149.7	28.7	127.3	28.7
H85	3765A × 5-27R	166.3	32.3	43.4	33.5
H86	3765A × LZ615R	140.7	34.2	55.5	23.7
H87	3765A × SCSR	154.0	27.7	66.5	24.8
H88	3765A × 0-30R	176.7	31.0	137.9	40.5
H89	3765A × R111	178.7	31.7	94.3	33.1
H90	3765A × L17R	200.0	28.0	70.7	32.3
H91	3765A × L2R	187.3	31.7	56.9	34.0
H92	3765A × J12R	117.7	32.0	60.2	21.8
H93	3765A × J105R	181.7	43.0	77.4	32.8
H94	3765A × XL7R	216.0	37.0	59.4	31.0
H95	3765A × JL5R	223.7	30.7	98.6	38.8
H96	3765A × 1383-2R	201.0	36.7	71.8	32.0
H97	3765A × 3560R	184.3	37.3	124.5	26.1
H98	3765A × JY15R	213.3	28.7	111.8	38.6
CK	Jinza 22	160.0	27.5	77.6	30.7
