# Supplementary material for: Identification of heterosis and combining ability in the hybrids of male sterile and restorer sorghum [Sorghum bicolor (L.) Moench] lines
Source: PLoS One. 2024 Jan 2;19(1):e0296416. doi: 10.1371/journal.pone.0296416 (PMC10760902; doi:10.1371/journal.pone.0296416)
Supplement: S4 Table — (PDF) [file pone.0296416.s007.pdf]

S4 Table. Estimates of mid-parent and better parent heterosis of 98 crosses for agronomic traits.

No.	Cross	MPH（%）				BPH（%）			
		Plant height	Panicle length	Grain weight per Panicle	1000-grain weight	Plant height	Panicle length	Grain weight per Panicle	1000-grain weight
H1	Tx3197A × 5-27R	29.49	7.8	-15.8	-3.22	23.2	2.93	-23.78	-9.94
H2	Tx3197A × LZ615R	49.71	13.08	25.95	-33.32	36.46	2.23	-10.94	-36.28
H3	Tx3197A × SCSR	40.34	4.98	21.56	29.62	27.67	-2.02	7.16	21.48
H4	Tx3197A × 0-30R	21.59	4.74	17.35	11.2	8.42	-1.38	-19.63	-0.42
H5	Tx3197A × R111	52.33	16.44	-0.36	14.66	35.54	11.78	-31.33	4.24
H6	Tx3197A × L17R	36.97	9.26	-13.33	1.97	21.74	6.86	-15.11	-14.13
H7	Tx3197A × L2R	36.87	-1.04	35.3	13.14	16.22	-3.4	51.52	12.28
H8	Tx3197A × J12R	15.01	4.4	4.56	2.83	10.14	-4.08	0.47	-0.57
H9	Tx3197A × J105R	44.04	-2.28	9.55	4.84	26.82	-17.61	0.89	-2.78
H10	Tx3197A × XL7R	45.9	8.43	7.3	4.68	31.69	5.24	-10.08	-10.1
H11	Tx3197A × JL5R	19.01	13.61	-20.81	12.14	-3.23	10.96	-35.4	4.63
H12	Tx3197A × 1383-2R	25.34	16.93	-20.96	5.67	6.24	11.2	-31.85	5.39
H13	Tx3197A × 3560R	27.04	0	-34.86	-7.95	6.05	-3.27	-40.57	-11.89
H14	Tx3197A × JY15R	37.59	22.54	-8.93	-10.99	17.75	14.54	-29.83	-16.24
H15	L407A × 5-27R	40.36	10.08	84.68	9.51	28.98	9.8	78.11	-4.75
H16	L407A × LZ615R	64.48	13.51	62.61	5.44	45.04	7.43	21.15	-6.02
H17	L407A × SCSR	62.26	16.19	44.94	20.26	42.82	3.66	53.76	19.2
H18	L407A × 0-30R	64.1	26.33	60.04	-0.68	41.7	13.66	15.62	-16.61
H19	L407A × R111	61.58	31.3	35.29	14.7	39.23	20.32	-1.93	-2.34
H20	L407A × L17R	29.38	5.65	16.18	-10.43	11.37	2.83	20.66	-28.9
H21	L407A × L2R	59.09	-0.72	15.99	11.73	31.08	-7.58	39.27	4.6
H22	L407A × J12R	37.41	2.84	6.3	0.83	27.07	-1	42.41	-3.22
H23	L407A × J105R	61.52	13.46	71.33	14.62	37.78	-0.25	66.39	14.34
H24	L407A × XL7R	66.4	32.85	57.32	-3.91	45.37	30.31	39.19	-22.34
H25	L407A × JL5R	59.71	20.03	19.08	20.7	26.26	16.99	1.55	5.24
H26	L407A × 1383-2R	57.15	20.95	26.4	15.16	29.27	20.75	13.89	6.77
H27	L407A × 3560R	62.08	26.69	33.94	15.7	31.39	24.69	28.94	12.18
H28	L407A × JY15R	77.14	16.96	40.28	6.67	47.05	4.5	13.25	-6.26
H29	A2V4A × 5-27R	34.41	12.2	27.43	-8.55	28.36	6.19	27.28	-10.53
H30	A2V4A × LZ615R	50.57	15.26	44.04	0.52	50.44	3.35	10.5	-0.08
H31	A2V4A × SCSR	58.18	23.1	36.38	17.23	57.97	15.89	26.86	4.75
H32	A2V4A × 0-30R	44.29	18.55	26.07	3.56	40.94	12.61	-6.17	-2.72
H33	A2V4A × R111	55.96	26.56	10.83	2.11	51.97	22.59	-17.35	-2.53
H34	A2V4A × L17R	25.6	11.62	34.43	-14.62	22.24	8.18	44.24	-24.86
H35	A2V4A × L2R	59.25	19.89	78.5	8.42	47.3	18.09	106.41	2.26

No.	Cross	MPH（%）				BPH（%）			
		Plant height	Panicle length	Grain weight per Panicle	1000-grain weight	Plant height	Panicle length	Grain weight per Panicle	1000-grain weight
H36	A2V4A × J12R	49.81	7.99	6.57	-2.29	42.14	-1.62	7.15	-10.08
H37	A2V4A × J105R	40.94	2.83	46.27	7.47	35.74	-13.95	46.33	-4.92
H38	A2V4A × XL7R	58.55	16.11	50.83	-7.64	56.97	11.69	37.55	-17
H39	A2V4A × JL5R	51.47	12.82	15.3	16.3	33.47	9.21	0.8	14.09
H40	A2V4A × 1383-2R	45.78	14.66	12.61	19.35	34.58	8.09	4.01	13.68
H41	A2V4A × 3560R	37.56	20.31	15.41	-4.88	24.84	15.35	14.48	-13.29
H42	A2V4A × JY15R	56.7	27.24	33.52	20.26	46.21	19.96	10.66	19.06
H43	1102A × 5-27R	27.02	11.61	41.05	-0.9	21.15	6.63	13.69	-2.31
H44	1102A × LZ615R	48.26	7.11	-18.77	10.85	47.93	5.8	-27.07	6.31
H45	1102A × SCSR	31.89	8.7	36.17	-6.38	31.89	-6.63	3.44	-18.95
H46	1102A × 0-30R	40.65	25	32.64	-17.5	37.57	8.23	15.86	-19.76
H47	1102A × R111	35.65	22.49	24.7	8.31	32.36	7.93	8.35	7.13
H48	1102A × L17R	29.3	4.12	15.01	-3	26	-2.82	-1.58	-11.83
H49	1102A × L2R	29.39	4.43	49.45	1.55	19.82	-6.59	36.16	-7.39
H50	1102A × J12R	43.38	6.13	17.21	-6.93	35.86	5.56	-5.06	-17.11
H51	1102A × J105R	36.77	6.8	25.22	-7.28	31.89	-2.37	15.93	-20.49
H52	1102A × XL7R	56.42	8.23	41.49	-13.89	55.06	1.75	28.68	-20.01
H53	1102A × JL5R	33.44	8.89	14.03	0.25	17.71	1.75	14.2	-1.45
H54	1102A × 1383-2R	39.78	10.85	-43.81	-15.8	29.2	6.33	-40.6	-22.49
H55	1102A × 3560R	33.28	15.19	17.02	-7.76	21.09	8.65	7.55	-18.59
H56	1102A × JY15R	51.19	20.82	25.72	-2	41.24	3.93	20.15	-4.53
H57	10480A × 5-27R	16.42	5	3.91	9.94	4.91	-7.47	-6.06	-6.5
H58	10480A × LZ615R	37.61	11.95	-27.34	-0.73	29.36	4.02	-28.47	-13.56
H59	10480A × SCSR	20.05	8.01	-13.95	-9.32	13.08	-13.53	-26.23	-12.45
H60	10480A × 0-30R	24.81	19.91	18.86	-12.2	20.09	-3.32	14.01	-27.84
H61	10480A × R111	29.51	21.93	23.09	15.98	24.92	-0.13	17.02	-3.39
H62	10480A × L17R	29.68	14.57	25.49	0.16	25.23	-1.15	19.75	-22.03
H63	10480A × L2R	18.65	21.06	-1.55	11.18	16.52	0.51	-0.56	1.53
H64	10480A × J12R	25.65	16.75	0.9	-6.58	12.54	6.73	-8.37	-12.58
H65	10480A × J105R	33.19	1.24	2.01	11.61	29.98	0.69	4.55	8.94
H66	10480A × XL7R	24.72	25.28	10.41	-15.92	18.46	8.81	11.34	-33.41
H67	10480A × JL5R	14.84	11.38	-19.12	-11.92	7.07	-3.8	-12.18	-24.91
H68	10480A × 1383-2R	30.69	7.86	-5.02	0.25	28.07	-4.59	8.81	-9.29
H69	10480A × 3560R	25.93	2.01	-9.75	-0.74	21.17	-11.14	-8.12	-6.2
H70	10480A × JY15R	33.23	9.83	17.78	4.37	32.06	-11.97	22.47	-10.35
H71	Tx623A × 5-27R	30.29	0.35	12.81	-1.49	26.53	-7.03	17.32	-2.04

No.	Cross	MPH（%）				BPH（%）			
		Plant height	Panicle length	Grain weight per Panicle	1000-grain weight	Plant height	Panicle length	Grain weight per Panicle	1000-grain weight
H72	Tx623A × LZ615R	55.2	7.57	24.46	-8.37	52.65	5.42	-1.24	-10.4
H73	Tx623A × SCSR	33.57	11.82	18.01	1.15	31.1	-6.5	32.48	-10.92
H74	Tx623A × 0-30R	38.95	26.22	36.83	14.62	33.45	6.35	5.4	9.35
H75	Tx623A × R111	45.67	17.45	29.68	-3.36	39.56	0.64	-0.06	-6.28
H76	Tx623A × L17	47.84	3.07	1.66	-13.86	41.47	-6.64	13.26	-23.09
H77	Tx623A × L2R	23.79	9.24	-0.83	6.2	12.69	-5.03	27.1	-1.36
H78	Tx623A × J12R	14.56	-0.63	0	-15.9	10.52	-4.28	20.96	-23.75
H79	Tx623A × J105R	41.5	7.71	7.41	-17.32	34.02	1.48	11.21	-27.88
H80	Tx623A × XL7R	43.77	14.83	25.06	-32.02	39.91	4.75	18.13	-38
H81	Tx623A × JL5R	35.38	13.73	31.07	8.54	17.51	3.14	17.96	8.24
H82	Tx623A × 1383-2R	39.97	1.3	7.95	-2.36	27.18	-5.78	2.61	-8.44
H83	Tx623A × 3560R	44.64	18.56	34.89	23.92	29.23	8.49	38.51	-0.57
H84	Tx623A × JY15R	44.42	8.4	54.11	-67.76	32.59	-9.24	31.66	-71.38
H85	3765A × 5-27R	35.58	18.78	-9.54	9.93	30.17	15.12	-28.15	4.63
H86	3765A × LZ615R	12.57	17.43	-19.63	-15.06	12.04	14.31	-30.18	-21.26
H87	3765A × SCSR	22.97	12.39	19.7	-12.85	22.11	-2.24	-10.6	-26.79
H88	3765A × 0-30R	36.15	17.22	40.77	10.42	32.27	2.79	18.92	9.52
H89	3765A × R111	45.87	18.28	20.65	5.22	41.37	5.58	1.53	2.63
H90	3765A × L17R	45.08	16.26	26.39	4.8	40.43	10.03	6.83	-1.52
H91	3765A × L2R	35.19	23.5	8.86	15.69	24.4	11.94	-1.82	2.16
H92	3765A × J12R	19.8	12.77	1.32	-12.82	14.27	11.71	-19.1	-24.74
H93	3765A × J105R	40.51	30.01	27.58	5.61	34.59	17.27	16.98	-12.08
H94	3765A × XL7R	69.11	36.34	-1.78	-8.03	66.49	30	-11.57	-11.6
H95	3765A × JL5R	47.06	17.88	15.13	11.54	28.96	11.7	12.05	5.87
H96	3765A × 1383-2R	42.87	25.88	-5.05	7.55	31.21	22.5	-2.44	-4.16
H97	3765A × 3560R	38.18	31.13	70.43	-7.6	24.77	25.44	55.11	-20.93
H98	3765A × JY15R	60.57	20.08	30.08	7.19	49.04	4.59	20.64	0.87

MPH, mid-parent heterosis; BPH, better parent heterosis
