# Supplementary material for: Identification of heterosis and combining ability in the hybrids of male sterile and restorer sorghum [Sorghum bicolor (L.) Moench] lines
Source: PLoS One. 2024 Jan 2;19(1):e0296416. doi: 10.1371/journal.pone.0296416 (PMC10760902; doi:10.1371/journal.pone.0296416)
Supplement: S5 Table — (PDF) [file pone.0296416.s008.pdf]

S5 Table. Estimates of standard heterosis of 98 crosses for agronomic traits.

No.	Crosses	SH（%）				
		Plant height	Panicle length	Grain weight per Panicle	1000-grain weight	Grain yield
H1	Tx3197A × 5-27R	-11.88	-0.73	-49.87	-11.40	-58.43
H2	Tx3197A × LZ615R	7.06	11.27	-8.76	-40.72	-44.14
H3	Tx3197A × SCSR	0.63	-13.82	-33.12	2.93	-52.42
H4	Tx3197A × 0-30R	-10.63	-13.45	-16.75	6.51	-41.70
H5	Tx3197A × R111	12.31	-1.82	-25.39	7.82	-34.62
H6	Tx3197A × L17R	1.13	-1.82	-44.20	6.19	-41.19
H7	Tx3197A × L2R	7.50	-15.27	-5.54	-4.89	-19.82
H8	Tx3197A × J12R	-22.31	0.73	-37.37	-15.96	-58.90
H9	Tx3197A × J105R	7.62	5.45	-21.91	-17.59	-60.92
H10	Tx3197A × XL7R	5.63	-1.82	-25.26	5.86	-28.34
H11	Tx3197A × JL5R	-0.19	2.18	-31.96	2.28	-25.83
H12	Tx3197A × 1383-2R	-1.31	8.36	-31.70	-10.42	-52.88
H13	Tx3197A × 3560R	2.31	-9.09	-53.99	-25.41	-51.27
H14	Tx3197A × JY15R	6.88	0.73	-25.90	-19.54	-23.61
H15	L407A × 5-27R	-7.75	6.55	17.14	-6.51	-39.32
H16	L407A × LZ615R	13.81	16.73	24.23	-12.70	-43.10
H17	L407A × SCSR	12.56	0.73	-14.69	-11.73	-54.39
H18	L407A × 0-30R	16.81	10.18	19.85	-10.75	-19.69
H19	L407A × R111	15.38	16.73	6.57	0.98	-26.23
H20	L407A × L17R	-7.50	-0.36	-20.62	-12.05	-46.25
H21	L407A × L2R	21.25	-10.18	-14.43	-12.70	-29.23
H22	L407A × J12R	-10.38	4.00	-32.22	-23.45	-60.05
H23	L407A × J105R	16.94	27.64	28.74	-16.94	-26.99
H24	L407A × XL7R	16.63	26.55	15.72	-8.47	-25.97
H25	L407A × JL5R	30.19	13.45	6.83	2.93	-39.66
H26	L407A × 1383-2R	20.06	17.45	14.18	-9.12	-32.09
H27	L407A × 3560R	26.75	21.09	-0.13	-13.03	-19.13
H28	L407A × JY15R	33.50	1.45	19.46	-10.10	-11.52
H29	A2V4A × 5-27R	0.88	2.55	-16.24	-12.05	-48.58
H30	A2V4A × LZ615R	18.25	12.36	13.27	-6.19	-39.90
H31	A2V4A × SCSR	24.50	0.00	-16.62	-1.63	-39.22
H32	A2V4A × 0-30R	16.19	-2.91	-2.71	4.23	-42.99
H33	A2V4A × R111	25.88	5.82	-10.18	0.65	-28.70
H34	A2V4A × L17R	1.50	-0.73	-5.15	-7.17	-41.08
H35	A2V4A × L2R	36.25	1.82	35.70	-3.91	26.03

No.	Crosses	SH（%）				
		Plant height	Panicle length	Grain weight per Panicle	1000-grain weight	Grain yield
H36	A2V4A × J12R	11.75	3.27	-29.64	-15.31	-52.99
H37	A2V4A × J105R	15.19	10.18	13.27	-10.75	-36.15
H38	A2V4A × XL7R	25.88	4.36	14.43	-2.28	15.44
H39	A2V4A × JL5R	37.63	0.73	6.06	11.40	-36.46
H40	A2V4A × 1383-2R	25.00	5.09	4.38	6.84	-25.37
H41	A2V4A × 3560R	20.44	8.36	-11.34	-18.57	-23.12
H42	A2V4A × JY15R	32.69	3.27	16.75	14.33	-15.20
H43	1102A × 5-27R	-4.50	13.09	11.60	-1.30	-58.61
H44	1102A × LZ615R	16.63	14.91	-25.26	7.49	-55.99
H45	1102A × SCSR	3.94	-1.09	1.55	-17.92	-40.70
H46	1102A × 0-30R	13.38	14.91	20.10	-14.01	26.81
H47	1102A × R111	9.63	14.55	17.78	10.75	7.57
H48	1102A × L17R	4.63	2.91	-3.48	9.12	-45.12
H49	1102A × L2R	10.81	-1.09	33.63	-6.19	33.64
H50	1102A × J12R	7.06	12.00	-6.83	-16.29	-52.11
H51	1102A × J105R	11.94	25.09	13.79	-19.54	-12.27
H52	1102A × XL7R	24.38	8.00	26.29	-5.54	-53.19
H53	1102A × JL5R	21.38	8.00	20.23	-0.33	-42.59
H54	1102A × 1383-2R	20.00	12.73	-40.46	-21.50	-41.63
H55	1102A × 3560R	16.81	15.27	5.54	-17.59	-26.92
H56	1102A × JY15R	28.19	10.18	26.80	-3.58	35.55
H57	10480A × 5-27R	-6.44	17.09	-8.63	-8.14	-42.92
H58	10480A × LZ615R	15.38	31.64	-26.68	-19.87	-46.72
H59	10480A × SCSR	0.81	9.45	-28.22	-35.18	-37.53
H60	10480A × 0-30R	7.06	22.55	18.17	-22.80	-20.60
H61	10480A × R111	11.38	26.55	27.19	0.00	8.59
H62	10480A × L17R	11.69	25.09	16.49	-3.58	19.69
H63	10480A × L2R	7.75	27.27	-3.22	-15.31	-30.40
H64	10480A × J12R	0.37	35.27	-10.82	-30.94	-43.94
H65	10480A × J105R	15.88	28.73	1.68	-21.17	-21.00
H66	10480A × XL7R	5.63	37.82	8.38	-21.50	-12.56
H67	10480A × JL5R	10.44	21.82	-7.60	-26.71	-17.09
H68	10480A × 1383-2R	18.94	20.73	9.15	-22.80	-32.18
H69	10480A × 3560R	16.88	12.36	-10.57	-27.36	-20.77
H70	10480A × JY15R	19.88	11.64	29.25	-14.01	17.67
H71	Tx623A × 5-27R	-3.94	5.09	-22.81	-3.58	-54.64

No.	Crosses	SH（%）				
		Plant height	Panicle length	Grain weight per Panicle	1000-grain weight	Grain yield
H72	Tx623A × LZ615R	19.81	19.27	1.16	-13.03	-37.68
H73	Tx623A × SCSR	3.31	5.82	-24.61	-13.36	-46.58
H74	Tx623A × 0-30R	10.00	20.36	9.28	16.94	-39.70
H75	Tx623A × R111	15.63	13.82	8.63	-2.93	6.63
H76	Tx623A × L17	17.50	5.82	-25.52	-4.89	-24.88
H77	Tx623A × L2R	4.25	7.64	-21.91	-4.23	-27.81
H78	Tx623A × J12R	-16.13	8.36	-31.19	-26.06	-68.57
H79	Tx623A × J105R	13.75	29.82	-13.92	-29.97	-32.51
H80	Tx623A × XL7R	12.25	18.55	-1.80	-27.04	7.94
H81	Tx623A × JL5R	21.19	16.73	24.10	5.86	-24.97
H82	Tx623A × 1383-2R	18.13	6.55	2.96	-11.07	-48.49
H83	Tx623A × 3560R	24.63	22.91	7.22	8.14	-25.34
H84	Tx623A × JY15R	20.38	2.91	38.92	-68.73	7.21
H85	3765A × 5-27R	1.19	18.55	-31.19	13.68	-48.56
H86	3765A × LZ615R	-12.06	24.36	-28.48	-14.33	-60.92
H87	3765A × SCSR	-3.75	0.73	-14.30	-20.52	-58.94
H88	3765A × 0-30R	9.00	5.82	23.20	19.22	-23.35
H89	3765A × R111	17.13	8.73	10.44	11.73	6.21
H90	3765A × L17R	16.63	13.09	2.32	21.82	-23.17
H91	3765A × L2R	15.06	15.27	-5.93	11.07	-20.50
H92	3765A × J12R	-11.19	17.09	-22.55	-18.24	-37.24
H93	3765A × J105R	14.25	50.18	12.11	-4.23	-29.30
H94	3765A × XL7R	33.56	33.82	-15.34	4.23	-45.23
H95	3765A × JL5R	33.00	14.91	17.91	15.31	-17.27
H96	3765A × 1383-2R	21.88	26.18	-2.19	4.23	-14.43
H97	3765A × 3560R	20.38	29.09	48.58	-14.01	29.50
H98	3765A × JY15R	35.25	7.64	27.32	9.77	18.51

SH，standard heterosis
