# Supplementary material for: Identification of heterosis and combining ability in the hybrids of male sterile and restorer sorghum [Sorghum bicolor (L.) Moench] lines
Source: PLoS One. 2024 Jan 2;19(1):e0296416. doi: 10.1371/journal.pone.0296416 (PMC10760902; doi:10.1371/journal.pone.0296416)
Supplement: Attachment — Submitted filename: Response to Editor.docx [file pone.0296416.s009.docx]

Dear Editor

We are glad to hear from you and have read the issues very carefully. The comments are very helpful in improving the quality and significance of the manuscript. Now I response the editor’s comment with a point by point and in blue the reply.

**1. We note that the grant information you provided in the ‘Funding Information’ and ‘Financial Disclosure’ sections do not match. Please see below for your reference.**

***Answer:*** We have ensured that our provided the updated Financial Disclosure Statement in the Author comments box or in the Cover Letter. Please update.

**2. It appears that your ORCiD iD has not been validated in your Editorial Manager account and we are unable to proceed until that step is complete.**

***Answer:*** The ORCiD iD has been modified according to the prompts, as shown in the following figure.

**3. Please ensure that you refer to Table 6 in your text as, if accepted, production will need this reference to link the reader to the Table.**

***Answer:*** We had ensured our refer to Table 6 in the manuscript. See at line 342 and 351 of page 18-19.

**4. Please include a copy of Table 4.**

***Answer:*** We had modified to Table 4 in the manuscript. See at line 290 of page 15.
